# Genetic analysis of HLA, NA and HPA typing in type 2 diabetes and ASO

**DOI:** 10.1111/j.1744-313X.2006.00581.x

**Published:** 2006-04

**Authors:** S Nomura, A Shouzu, S Omoto, T Matsuzaki, M Yamaoka, M Abe, M Hosokawa, M Nishikawa, T Iwasaka, S Fukuhara

**Affiliations:** * The First Department of Internal Medicine; † The Second Department of Internal Medicine; ‡ Department of Blood TransfusionKansai Medical University, Osaka, Japan

## Abstract

We examined the genetic status of human leucocyte antigens (HLA), human platelet alloantigens (HPA) and neutrophil-specific antigens (NA) in patients with type 2 diabetes mellitus and diabetic arteriosclerosis obliterans (ASO). To our knowledge, the present study is the first report showing the relationship among three genetic factors in type 2 diabetes mellitus and ASO patients. HLA typing was performed by the polymerase chain reaction (PCR)–restriction fragment length polymorphism method. HPA-typing and NA-typing were by a PCR-sequence-specific primer method. The incidence of HLA-DRB1^*^ 1501 was found to be significant in type 2 diabetes and non-diabetic, particularly ASO-positive patients, compared to control subjects. There were no differences in NA1/NA2 between the control and diabetic or non-diabetic ASO groups. However, the frequency of NA2/NA2 in ASO-positive diabetes and non-diabetic ASO patients was significantly higher than controls. The a/b genotype of HPA-5a/5b was significantly lower in type 2 diabetes and non-diabetic ASO-positive patients than in controls. These findings suggest that genetic studies of HLA, NA and HPA could be useful to understand the pathogenesis of type 2 diabetes and ASO.

## Introduction

The underlying pathology of atherosclerosis obliterans (ASO) is basically atherosclerosis, and complete occlusions by fresh or old thrombi are often observed (Schwartz *et al*., 1995; Williams & Tabas, 1995). Recent evidence indicates that the incidence of thrombotic disease is steadily increasing among Asian populations (Kauffmann-Zeh *et al*., 2000). Epidemiological studies indicate that thrombotic diseases result from complex interactions between susceptibility factors, chronic environmental influences and established intercurrent disorders such as diabetes mellitus (Kunicki, 2002). The critical role of platelets in the thrombotic process is now well accepted (Rauch *et al*., 2001). At sites of vascular injury, thrombus formation is initiated by binding platelet glycoprotein (GP) Ib to von Willebrand factor and binding platelet GPIa/IIa to collagen, which leads to platelet activation and aggregation by binding platelet GPIIb/IIIa to fibrinogen and von Willebrand factor (Kunicki, 2000). Several polymorphisms in the genes encoding for platelet GPs have been associated with increased platelet adhesiveness and aggregation, and in some studies, they have been associated with an increased risk of arterial thrombosis (Bray, 1999; Bussel *et al*., 2000; Reiner *et al*., 2000). Human platelet alloantigens (HPA) are important for sensitization to platelet GP polymorphisms. In general, HPAs play important roles in the pathogenesis of neonatal alloimmune thrombocytopenia and post-transfusion purpura, as well as in some cases of refractoriness to platelet transfusion (Von dem Borne Kr. *et al*., 1981; Von dem Borne Kr. & Ouwehand, 1989; McFarland *et al*., 1991; Dreyfus *et al*., 1997). However, there are few reports available concerning the relationship between type 2 diabetes and diabetic ASO.

Leucocytes have been suggested to play an important role in the development of atherosclerosis (Beekhuizen & van Furth, 1993; Jonasson *et al*., 1986). The first step in the process of leucocyte infiltration into the sub-endothelial space is adhesion of circulating cells to the endothelium (Jonasson *et al*., 1986). Thus, the pathogenesis of atherosclerosis appears to be inflammation (Ross, 1993). A recent study suggests genetic predisposition and chronic inflammation play the leading roles in the early stages and in the development of vascular damage (Diamantopoulos *et al*., 2002; Tsuchiya *et al*., 2003). In addition, the Fcγreceptor polymorphism is related to susceptibility of some immunological diseases (Dijstelbloem *et al*., 2000; Nieto *et al*., 2000; Fujimoto *et al*., 2001), and neutrophil-specific antigens (NA)1 and NA2 are located on the Fcγreceptor IIIb (Matsuo *et al*., 2000). On the other hand, studies have long suggested associations between certain human leucocyte antigens (HLA) and many autoimmune diseases. With respect to the genetic aspects of thrombotic diseases such as type 2 diabetes mellitus, the HLA haplotype of patients can possibly be considered a potentially important factor in the aetiology, although its role remains unclear.

This study examined the genetic status of HLA, HPA and NA in patients with type 2 diabetes mellitus and diabetic ASO. To our knowledge, this is the first report detailing relationships among these three genetic factors in type 2 diabetes mellitus and ASO patients.

## Materials and methods

### Subjects

We studied 104 Japanese patients (67 men and 37 women, aged 39–85 years) with type 2 diabetes mellitus, as defined by the American Diabetes Association Criteria (The Expert Committee on the Diagnosis and Classification of Diabetes Mellitus, 1997). Seventeen non-diabetic ASO patients were used for patient control subjects. A brief clinical profile of the 104 diabetic patients and 17 non-diabetic ASO patients is presented in Table 1. One hundred and twenty-nine controls were randomly selected from among healthy unrelated Japanese individuals. Approval was obtained from the Institutional Review Board for these studies, and informed consent was obtained in accord with the Declaration of Helsinki.

### Diagnosis of ASO

All patients were evaluated by the ankle-pressure index (API). Patients with an API > 1.0 were considered not to have ASO. Thirty-three patients with API < 1.0 underwent angiography and plethysmography, and 28 were diagnosed as having ASO (Fontain's class I–III). Of these, 21 patients with ASO underwent bypass procedures for femoral and trifurcation occlusive disease. Seventeen non-diabetic ASO patients were also diagnosed with Fontain's class I–III. Of these, 10 patients with ASO underwent bypass procedures.

### HLA DNA typing by PCR–RFLP

Genomic DNAs from 104 diabetic patients, 17 non-diabetic patients and 126 healthy control subjects were isolated by phenol extraction of sodium dodecyl sulphate-lysed and proteinase K-treated cells. DNA was amplified by PCR with *Taq* DNA polymerase, and HLA typing was performed by the PCR–restriction fragment length polymorphism (RFLP) method (Ota *et al*., 1992). The reaction mixture was subjected to 30 cycles of denaturation at 96–97 °C for 1 min, annealing at 55–62 °C for 1 min, and extension at 72 °C for 2 min in an automated PCR thermal sequencer (Iwaki Glass, Inc., Tokyo, Japan). After amplification, aliquots of the products were digested with allele-specific restriction endonucleases for 3 h after addition of the appropriate reaction buffer. Samples of the amplified and cleaved DNAs were subjected to electrophoresis on 12% polyacrylamide gel in a minigel apparatus (Mupid-2, Cosmo Bio Co., Ltd, Tokyo, Japan). Cleavage of amplified fragments was detected by staining with ethidium bromide, and genotypes were determined on the basis of the RFLP band patterns thus generated.

**Table 1:** Clinical characteristics of the diabetic and non-diabetic patients

	Type 2 DM		Non-diabetic
		
	ASO (–)	ASO (+)	ASO
No. of patients	76	28	17
Sex ratio (male : female)	51 : 25	22 : 6	13 : 4
Age (year)			
Range	39–81	52–85	47–66
Median	54	66	59
BMI (kg m^−2^)			
Mean ± S.E.	28.1 ± 1.6	28.3 ± 2.1	24.2 ± 1.7

### NA genotyping by PCR–preferential homoduplex formation assay (PHFA)

We applied PCR–preferential homoduplex formation assay (PHFA) developed by Oka *et al*. (1994) to DNA typing of neutrophil-specific antigens (NA1, NA2 and SH) located on the neutrophil receptor, FcγRIIIb. Gene fragments containing the polymorphic sequences corresponding to NA1, NA2 and SH of FcγRIIIb, as well as the homologous fragment of FcγRIIIa, were amplified by PCR using specific primers (Fujiwara *et al*., 1996, 1999). Assay procedure was performed according to the manufacturer's instructions (Wakunaga Pharm. Co., Ltd, Hiroshima, Japan).

### HPA DNA typing by enzyme-linked mini-sequence assay (ELMA)

Gene fragments that contained polymorphic sequences corresponding to HPA-1, HPA-2, HPA-3, HPA-4, HPA-5 and HPA-6 were specifically amplified by the PCR method. Enzyme-linked mini-sequence assay (ELMA) typing was performed according to the manufacturer's instructions (Smitest HPA Genotype ELMA, Sumitomo Metal Industries, Ltd, Ibaraki, Japan).

### Statistical analysis

The χ^2^ method with continuity correction and Fisher's exact test were used for HLA data analysis. Relative risk was calculated according to Wolf's method with Holdane's correction. Briefly, it was calculated as (a × d)/(b × c), where a, b, c and d are the number of marker-positive patients, marker-negative patients, marker-positive controls and marker-negative controls, respectively (Thomson, 1981). The frequencies of the FcγRIIIb and HPA alleles were estimated by a gene-counting method and tested for deviation from the Hardy–Weinberg equilibrium using the χ^2^ test.

**Figure 1:**
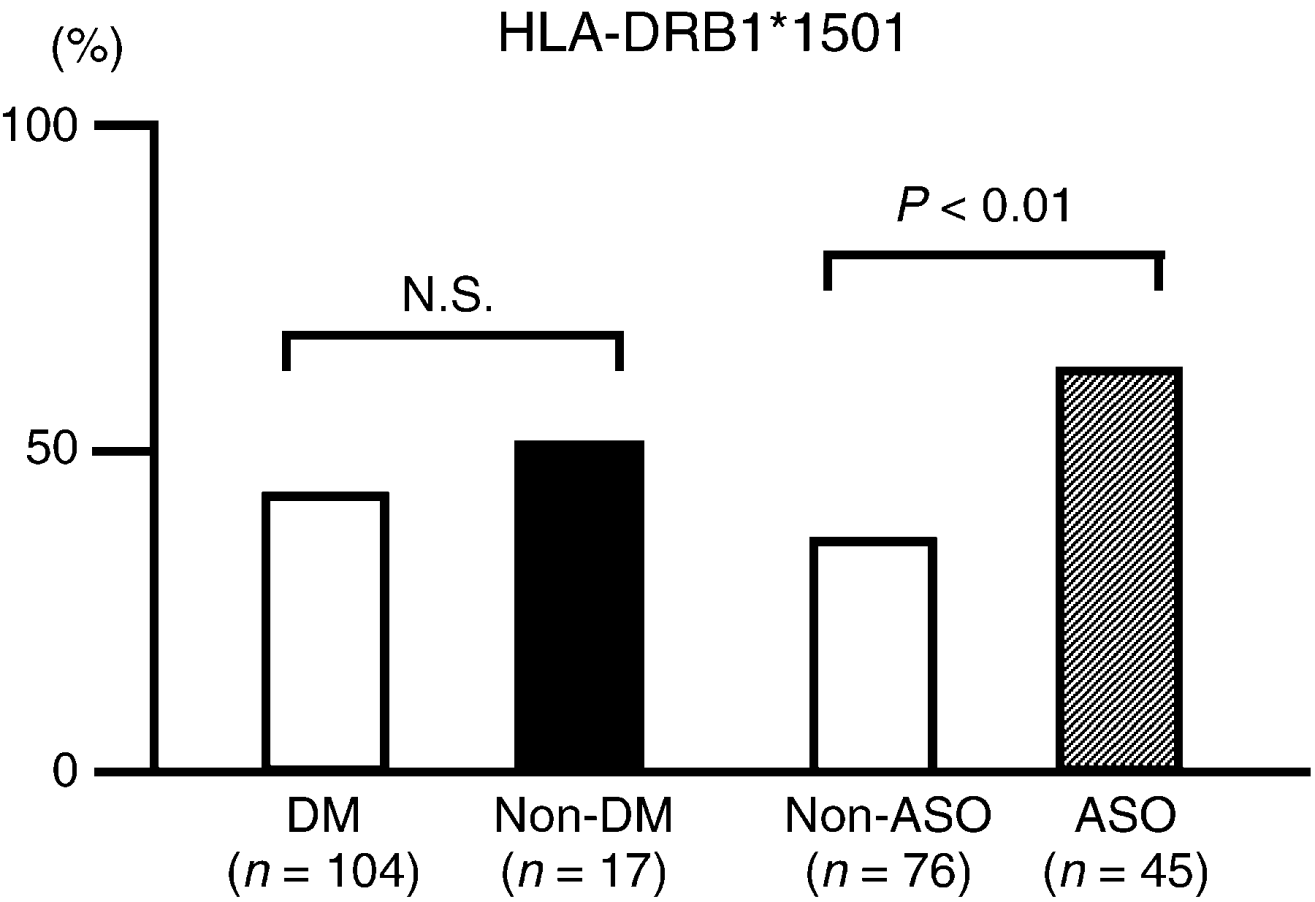
Relative frequencies of HLA-DRB1*1501 in type 2 diabetes and ASO patients. N.S. not significant.

## Results

Table 2 shows the HLA-DRB1 alleles in the control and type 2 diabetes patients. The incidence of HLA-DRB1*1501 was found to be significant in type 2 diabetes and non-diabetic ASO-positive patients compared to controls (control vs. ASO-negative diabetic patients, 11.6% vs. 35.5%, *P* < 0.05; control vs. ASO-positive diabetic patients, 11.6% vs. 60.7%, *P* < 0.01; control vs. non-diabetic ASO-positive patients, 11.6% vs. 52.9%, *P* < 0.05). In addition, the incidence of this allele was found to be significant in ASO patients compared to non-ASO patients, although there was no significant difference between diabetic and non-diabetic patients (Fig. 1). On the other hand, another DRB1 allele did not exhibit significant differences.

**Table 2:** Frequencies of HLA-DRB1 alleles

	Healthy	Type 2 DM (%)		Non-diabetic
	Controls (%)			
DRBI	*n* = 129	ASO (–) *n* = 76	ASO (+) *n* = 28	ASO *n* = 17
0101	17.8	13.2	10.7	11.8
0301	0.8	0	0	0
0401	0.8	0	0	0
0403	1.6	0	0	0
0404	0.8	0	0	0
0405	20.2	38.2	21.4	23.5
0406	13.2	5.3	3.6	5.9
0407	1.6	0	0	0
0410	4.7	2.6	3.6	5.9
0802	14.0	6.6	3.6	5.9
0803	17.8	19.7	17.9	23.5
0804	2.3	1.3	3.6	0
0901	20.2	22.4	17.9	17.6
1001	0.8	0	0	0
1101	2.3	5.3	3.6	5.9
1201	5.4	1.3	0	0
1202	0	2.6	0	0
1301	3.9	1.3	3.6	0
1302	11.6	6.6	7.1	11.8
1401	4.7	1.3	3.6	5.9
1402	0.8	0	0	0
1403	3.9	2.6	0	0
1405	4.7	1.3	0	0
1406	3.1	1.3	3.6	0
1407	0.8	0	0	0
1501	11.6	35.5^a^	60.7^b^	52.9^c^
1502	21.7	19.7	17.9	17.6
1601	1.6	1.3	3.6	5.9
1602	2.3	3.9	3.6	5.9

DM: diabetes mellitus.

ASO: arteriosclerosis obliterans.

a *P* < 0.05 (χ^2^ = 4.187).

b *P* < 0.01 (χ^2^ = 11.745).

c *P* < 0.05 (χ^2^ = 5.642).

Table 3 shows NA gene frequency in control and type 2 diabetes patients; there were no differences in NA1/NA2 among the control, type 2 diabetes and non-diabetic ASO groups. However, the frequency of NA2/NA2 in ASO-positive diabetic and non-diabetic ASO patients was significantly higher than in controls (control vs. ASO-negative diabetic patients, 12.4% vs. 23.7%, N.S.; control vs. ASO-positive diabetic patients, 12.4% vs. 39.3%, *P* < 0.05; control vs. non-diabetic ASO-positive patients, 12.4% vs. 41.2%, *P* < 0.05).

**Table 3:** Results of genotyping of FcγRIIIB in healthy controls and patients

	Healthy	Type 2 DM		Non-diabetic
	controls			
Genotypes	*n* = 129	ASO (–) *n* = 76	*ASO (+) n* = 28	ASO *n* = 17
NA1/NA1	48 (37.2%)	22 (28.9%)	3 (10.7%)	2 (11.7%)
NA1/NA2	65 (50.4%)	36 (47.4%)	14 (50.0%)	8 (47.1%)
NA2/NA2	16 (12.4%)	18 (23.7%)	11 (39.3%)^a^	7 (41.2%)^b^

a *P* < 0.05 (χ^2^ = 4.570).

b *P* < 0.05 (χ^2^ = 4.892).

Table 4 shows the HPA genotypes of the control and diabetic patients. The a/b genotype of HPA-5a/5b was significantly lower in type 2 diabetes and non-diabetic ASO patients than in controls (Control vs. ASO-negative diabetic patients, 7.0% vs. 1.3%, N.S.; control vs. ASO-positive diabetic patients, 7.0% vs. 0%, *P* < 0.05; control vs. non-diabetic ASO-positive patients, 7.0% vs. 0%, *P* < 0.05).

## Discussion

Polymorphisms of class II genes within the major histocompatibility complex can now be precisely determined by typing using the PCR–RFLP method. For example, serologically defined HLA-DR antigens can be divided into many alleles reflecting the polymorphism of the amino acid sequence in the allelic hypervariable regions of the β chain. In addition, it has been reported that aplastic anemia patients with HLA-DR2 are likely to respond to immunosuppressive therapy (Nimer *et al*., 1994; Nakao *et al*., 1994). We have reported that the frequency of HLA-DR decreased and that of DRB1*0410 significantly increased in immune thrombocytopenic purpura patients with poor response to prednisolone (Nomura *et al*., 1998). Thus, the analysis of HLA antigens and alleles appears to offer some guidelines for the treatment of autoimmune diseases. In the present study, HLA DRB1*1501 was found to be more common in diabetic, particularly ASO-positive, patients. In addition, this allele was also found to be more common in non-diabetic ASO-positive patients. Thus, the association between patients and DRB1*1501 is not always related to type 2 diabetes, because this association is detected in non-diabetic patients. Type 1 diabetes was reported to be associated with some HLA alleles (Park, 2004). However, for the type 2 allele, such relationship was not detected. On the other hand, the association between HLA and infection is possible to detect. In the recent study, Veneri *et al*. (2005) reported the relationship between *Helicobacter pylori* and HLA in immune thrombocytopenic purpura patients. Our findings suggest the hypothesis that HLA regulates immunoreactive inflammation or infection associated with type 2 diabetes or ASO. Thus, analysis of some HLA alleles may provide the information for predicting ASO or type 2 diabetes, if racial differences are taken into account. Diamantopoulos *et al*. (2002) evaluated 197 patients with type 2 diabetes, and found a significant association between HLA-A3 and carotid intimal media thickness. In the Japanese population, the frequency of HLA-A3 is extremely low. In addition, most DRB1*1501 are associated with HLA-A2, -A24 or -A26. The discrepancy between our results and Diamontopoulos's is thought to depend on racial differences. However, most autoimmune diseases share pathogenetic mechanisms characterized by association with an HLA class II haplotype. Also, the pathogenesis of diabetic or non-diabetic ASO may be heterogeneous; therefore, analysis of a larger population with diverse genetic backgrounds will be needed.

**Table 4:** Genotype frequencies of the six major HPAs

		Type 2 DM		Non-diabetic
	Healthy			
Genotype	controls *n* = 129	ASO (–) *n* = 76	*ASO (+) n* = 28	ASO *n* = 17
HPA-1a/1a (Pl^A1^/Pl^A1^)	100 (%)	100 (%)	100 (%)	100 (%)
HPA-1a/1b (Pl^A1^/Pl^A2^)	0	0	0	0
HPA-1b/1b (Pl^A2^/Pl^A2^)	0	0	0	0
HPA-2a/2a (Ko^b^/Ko^b^)	73.6	84.2	82.1	82.4
HPA-2a/2b (Ko^b^/Ko^a^)	23.3	14.5	17.9	17.6
HPA-2b/2b (Ko^a^/Ko^a^)	3.1	1.3	0	0
HPA-3a/3a (Bak^a^/Bak^a^)	34.9	26.3	35.7	35.3
HPA-3a/3b (Bak^a^/Bak^b^)	51.2	57.9	46.4	41.2
HPA-3b/3b (Bak^b^/Bak^b^)	14.0	15.8	17.9	23.5
HPA-4a/4a (Pen^a^/Pen^a^)	98.4	97.4	96.4	94.1
HPA-4a/4b (Pen^a^/Pen^b^)	1.6	2.6	3.6	5.9
HPA-4b/4b (Pen^b^/Pen^b^)	0	0	0	0
HPA-5a/5a (Br^b^/Br^b^)	93.0	98.7	100	100
HPA-5a/5b (Br^b^/Br^a^)	7.0	1.3*	0**	0***
HPA-5b/5b (Br^a^/Br^a^)	0	0	0	0
HPA-6a/6a (Ca^a^/Ca^a^)	96.9	96.1	96.4	100
HPA-6a/6b (Ca^a^/Ca^b^)	3.1	3.9	3.6	0
HPA-6b/6b (Ca^b^/Ca^b^)	0	0	0	0

* *P* < 0.05 (χ^2^ = 5.625);

** *P* < 0.05 (χ^2^ = 4.492);

*** *P* < 0.05 (χ^2^ = 4.314).

FcγRIIIb is a glycosylphosphatidylinositol-anchored protein expressed on neutrophils (Ravetch & Perussia, 1989). NA1 and NA2 located on the neutrophil receptor FcγRIIIb are known to be involved in disorders such as neonatal alloimmune neutropenia, autoimmune neutropenia and transfusion-related acute lung injury (Lalezari *et al*., 1960, 1975; Yomtovian *et al*., 1984). In addition, polymorphisms influencing the binding affinity between the FcγRs and IgG of different subclasses are recently thought to be important for individual susceptibility to infection with gram-negative bacteria contributing to periodontal disease (Meisel *et al*., 2001). Indeed, Kobayashi *et al*. (2000) reported the importance of the FcγRIIIb-NA2 allele for susceptibility to IgG in Japanese periodontitis patients. In the present study, the frequency of NA2/NA2 was found to be significantly higher in ASO-positive type 2 diabetes and non-diabetic ASO patients than in controls. Our results suggest the importance of the NA2 allele in susceptibility to ASO. Apart from some reports of periodontal disease, Fijen *et al*. (2000) reported that the FcγRIIIb-NA2/NA2 allotype appeared associated with meningococcal disease. However, previous reports have not always noted the importance of the NA2 allele. For example, Raknes *et al*. (1998) reported that myasthenia gravis patients with the NA1/NA1 genotype had the most severe condition. The function of FcγRIIIb is not entirely clear; however, interestingly, FcγRIIIb induces actin filament assembly that is a prerequisite for phagocytosis, while cross-linking of the FcγRIIIb by immune complexes enhances FcγRII mediated phagocytosis (Salmon *et al*., 1991; Flesch *et al*., 1998). These functions may be related to the development of atherosclerosis, since the pathogenesis of atherosclerosis includes inflammation. In addition, our results possibly reflect on such a hypothesis.

HPA plays an important role in provoking neonatal alloimmune thrombocytopenic purpura, post-transfusion purpura and refractoriness to platelet transfusion. To date, 10 HPA systems have been described and several additional alloantigen systems have been reported (Bussel *et al*., 2000; Kunicki, 2000, 2002). In particular, HPA-1 (Pl^A.^), HPA-2 (Ko), HPA-3 (Bak), HPA-4 (Pen) and HPA-5 (Br) have been well established by serological analysis (Kunicki & Newman, 1992). The HPA-1a (Pl^A1^, Zw^a^) antigen is by far the most common antigen implicated in neonatal alloimmune thrombocytopenia in a Caucasian population (Taaning *et al*., 1983; McFarland *et al*., 1989; Mueller-Eckhardt *et al*., 1989). On the other hand, HPA-4b is responsible for neonatal alloimmune thrombocytopenia in Japanese. This discrepancy in findings would have occurred because the HPA phenotype frequency varies between races. HPA systems also reside on functional platelet receptor proteins. It has been shown by a number of groups that all platelet-specific alloantigens thus far discovered are located on four platelet membrane GPs: GPIIb, GPIIIa, GPIb and GPIa. Thus, it has been reported that polymorphism of HPA is a genetic risk factor for thrombotic diseases (Weiss *et al*., 1996; Gonzalez-Conejero *et al*., 1998; Sperr *et al*., 1998; Carter *et al*., 1999; Kroll *et al*., 2000; Baker *et al*., 2001; Mikkelsson *et al*., 2001, 2002; Jilma-Stohlawetz *et al*., 2003). In the present study, the a/b genotype of HPA-5a/5b was significantly lower in type 2 diabetes and non-diabetic ASO patients than in controls. The Lys_505_Glu amino acid substitution located in the cation-binding domain of α_2_ is responsible for HPA-5b (Br^a^) and HPA-5a (Br^b^) epitope formation (Santoso *et al*., 1993). In a large study, Kroll *et al*. (2000) found an association between HPA-5 dimorphism in a low-risk patient subgroup with coronary artery disease. In this population, the frequency of Br^b^ homozygous individuals was overrepresented. This finding suggests that the allele 3 (807C; Br^a^) may increase the risk for thrombotic disease through a quantitative effect on the function that is independent of the genetic effects on expression levels. However, the frequency of phenotype HPA-5b in Japanese is lower than that in Caucasian (8.7 vs. 20.6%). In addition, it has been reported that the probability of HPA-related immunization is strongly associated with HLA class II DR types (Gonzalez-Conejero *et al*., 1998). A larger number of patients will need to be examined to confirm this possibility.

In conclusion, we performed a genetic analysis of HLA, NA and HPA in Japanese control subjects and type 2 diabetes patients with and without ASO. The incidence of HLA-DRB1*1501 was found to be significant in type 2 diabetes and non-diabetic ASO-positive patients compared with controls, and the frequency of this allele was significant in patients with ASO. The frequency of NA2/NA2 in ASO-positive diabetes and non-diabetic ASO-positive patients was significantly higher than in controls. The a/b genotype of HPA-5a/5b was significantly lower in type 2 diabetes and non-diabetic ASO-positive patients than in controls. These findings suggest that genetic studies of HLA, NA and HPA could be useful to understand the pathogenesis of type 2 diabetes and ASO.
